# Supplementary material for: A Likelihood Approach for Real-Time Calibration of Stochastic Compartmental Epidemic Models
Source: PLoS Comput Biol. 2017 Jan 17;13(1):e1005257. doi: 10.1371/journal.pcbi.1005257 (PMC5240920; doi:10.1371/journal.pcbi.1005257)
Supplement: S1 File — (TAR.GZ) [file pcbi.1005257.s014.tar.gz › HSPH_Online-SI-Revision/MSS10/n10-mild/n10-mild_NEW_table-8.pdf]

1	R0	{1.25046, 6.80278, 0.1}	{1.15825, 2.78738, 0.13}	{{1.06738, 2.19254, 0.}, {1.25474, 3.26445, 0.}}	{10.1694, 64.9573, 216.}	inf	59.	35.7248	{6.43035, 61.5324}	52.442	0.386481	newly	76.	105.59	{14., 210.}	74.6184	0.310095	Reff	1.22245	1.13478	{1.04494, 1.22918}	9.98543	ar	3516.	2125.37	{223., 3787.}	49.688
2	R0	{1.32013, 4.45847, 0.1}	{2.48946, 14.0671, 0.72}	{{1.93539, 9.82151, 0.}, {2.98558, 18.1089, 1.}}	{88.5772, 215.515, 688.}	inf	43.	150.016	{94.9549, 212.647}	248.875	0.525519	newly	76.	109.22	{81., 139.}	44.9474	0.153764	Reff	1.30046	2.42448	{1.89951, 2.88756}	86.4326	ar	4420.	8790.24	{7914., 9382.}	98.8742
3	R0	{1.31785, 5.36796, 1.}	{1.36856, 3.3501, 0.03}	{{1.22544, 2.19254, 0.}, {1.53501, 4.06661, 0.}}	{11.3475, 37.9827, 99.}	inf	49.	45.0179	{12.6572, 77.839}	46.3756	0.257908	newly	66.	142.22	{38., 236.}	132.	0.338742	Reff	1.29953	1.34989	{1.20816, 1.51284}	11.2406	ar	4942.	4560.96	{2481., 6032.}	26.5463
4	R0	{1.38801, 6.16031, 0.1}	{1.69712, 9.66915, 1.16}	{{1.06738, 2.19254, 0.}, {2.31174, 17.2836, 2.}}	{36.3792, 103.87, 1142.}	inf	59.	111.798	{15.6157, 188.132}	125.378	0.409137	newly	85.	105.77	{43., 161.}	42.7176	0.177587	Reff	1.35636	1.64639	{1.04569, 2.22701}	35.3379	ar	4790.	5687.81	{918., 8618.}	63.1885
5	R0	{1.27955, 11.57, 1.}	{1.13388, 5.78493, 0.05}	{{1.13227, 5.71232, 0.}, {1.13726, 6.0871, 0.}}	{11.3842, 50.0006, 95.}	inf	102.	55.9112	{37.0097, 73.084}	45.1851	0.275759	newly	64.	70.28	{47., 94.}	25.7813	0.106208	Reff	1.19011	1.05985	{1.0559, 1.06376}	10.9452	ar	3460.	2100.79	{1445., 2644.}	39.2835
6	R0	{1.22184, 4.34395, 1.}	{1.65536, 4.54823, 0.17}	{{1.22544, 2.5364, 0.}, {2.45236, 6.25659, 1.}}	{37.0096, 30.926, 91.}	inf	20.	19.1289	{3.8705, 38.1533}	54.7446	0.30576	newly	28.	50.02	{11., 91.}	108.571	0.348682	Reff	1.21683	1.64844	{1.22116, 2.43103}	36.9881	ar	3524.	5540.8	{727., 8891.}	79.66
7	R0	{1.23856, 4.59068, 0.1}	{2.57235, 15.5271, 2.09}	{{2.19696, 12.0512, 2.}, {2.99088, 18.9406, 3.}}	{107.689, 238.232, 1992.}	inf	52.	282.738	{190.915, 383.84}	443.726	0.718872	newly	78.	177.7	{128., 219.}	127.821	0.349751	Reff	1.20995	2.45148	{2.1071, 2.83279}	102.61	ar	3855.	8915.86	{8395., 9400.}	131.28
8	R0	{1.34531, 10.6689, 1.}	{1.20506, 3.27268, 0.24}	{{1.06738, 2.19254, 0.}, {1.28596, 3.98513, 1.}}	{10.4251, 69.325, 82.}	inf	98.	45.8595	{16.4759, 78.1757}	53.4134	0.398278	newly	72.	115.59	{40., 193.}	80.3472	0.258113	Reff	1.29352	1.16491	{1.03402, 1.24363}	9.9422	ar	4852.	2990.66	{1225., 4064.}	38.3623
9	R0	{1.31775, 5.19226, 0.1}	{2.18982, 12.8726, 0.82}	{{1.87927, 9.63533, 0.}, {2.64334, 17.2462, 2.}}	{66.1789, 147.92, 784.}	inf	51.	142.982	{91.6067, 208.055}	180.357	0.427427	newly	85.	106.18	{77., 134.}	29.3647	0.108565	Reff	1.29522	2.13105	{1.83518, 2.55386}	64.5324	ar	4708.	8283.53	{7580., 9085.}	75.9458
10	R0	{1.25916, 6.89207, 0.1}	{1.15476, 2.8243, 0.19}	{{1.06738, 2.19254, 0.}, {1.25474, 3.57392, 1.}}	{8.58947, 59.021, 252.}	inf	121.	63.287	{19.7042, 121.016}	49.8118	0.395094	newly	119.	166.83	{64., 313.}	66.2437	0.259324	Reff	1.17404	1.08332	{0.996776, 1.18044}	8.16572	ar	3823.	2479.61	{958., 3819.}	36.0991
11	R0	{1.20377, 5.46566, 0.1}	{1.3693, 1.97101, 0.02}	{{1.24751, 1.89883, 0.}, {1.39162, 2.5364, 0.}}	{14.2023, 63.9382, 118.}	inf	61.	73.857	{11.793, 155.064}	75.8828	0.350907	newly	77.	488.94	{84., 1060.}	542.753	0.68456	Reff	1.17392	1.33352	{1.22078, 1.36359}	14.0376	ar	3114.	4699.53	{3773., 5283.}	57.2303
12	R0	{1.27878, 2.55804, 0.1}	{2.29547, 9.27877, 0.95}	{{1.70301, 4.00571, 0.}, {2.95376, 15.5722, 2.}}	{79.9333, 264.767, 944.}	inf	6.	45.3941	{17.2119, 71.5422}	657.926	0.820202	newly	12.	71.28	{27., 121.}	495.333	0.689051	Reff	1.27328	2.27578	{1.68435, 2.92491}	79.1705	ar	3930.	8126.49	{6839., 9382.}	111.028
13	R0	{1.41227, 5.45696, 0.1}	{1.71398, 16.3661, 2.86}	{{1.47332, 12.3431, 3.}, {1.87294, 19.0269, 3.}}	{22.9443, 203.502, 2768.}	inf	41.	122.915	{69.7861, 167.238}	201.333	0.461073	newly	62.	62.68	{44., 80.}	20.3226	0.0853458	Reff	1.38233	1.66302	{1.43798, 1.81139}	21.8979	ar	4822.	6786.11	{5565., 7645.}	45.9044
14	R0	{1.32917, 7.27593, 1.}	{1.90069, 15.5757, 2.56}	{{1.38815, 6.71734, 0.}, {2.1968, 19.9579, 3.}}	{43.443, 120.023, 182.}	inf	80.	186.006	{79.5459, 231.454}	138.43	0.367881	newly	96.	102.13	{80., 122.}	14.7604	0.0622218	Reff	1.28969	1.82279	{1.34674, 2.10084}	41.7532	ar	4758.	7445.21	{5135., 8409.}	58.3894
15	R0	{1.31629, 9.97481, 2.}	{1.71879, 9.77485, 0.95}	{{1.22544, 3.07157, 0.}, {2.45321, 19.391, 2.}}	{34.7472, 67.0698, 53.5}	inf	84.	110.095	{18.3696, 225.858}	86.8997	0.379896	newly	70.	99.6	{39., 145.}	59.0571	0.229752	Reff	1.2843	1.66831	{1.20106, 2.36152}	33.6548	ar	4543.	5855.13	{3242., 8868.}	50.2036
16	R0	{1.36735, 5.74166, 0.1}	{1.21855, 3.08531, 0.2}	{{1.06738, 2.19254, 0.}, {1.28596, 3.98513, 1.}}	{11.5374, 46.2644, 272.}	inf	40.	32.8353	{9.50996, 59.0944}	41.6981	0.263635	newly	65.	94.55	{26., 180.}	77.0308	0.277762	Reff	1.34356	1.19818	{1.04947, 1.26512}	11.4513	ar	4516.	3019.1	{526., 4192.}	35.2068
17	R0	{1.27524, 13.2396, 0.1}	{1.73948, 15.6287, 0.49}	{{1.40261, 9.98152, 0.}, {1.8951, 19.1994, 1.}}	{36.4038, 31.5938, 492.}	inf	39.	34.2082	{14.7336, 51.7336}	31.7227	0.17524	newly	23.	18.14	{9., 29.}	33.4783	0.191502	Reff	1.26504	1.72613	{1.39379, 1.87935}	36.449	ar	3731.	6888.53	{5021., 7737.}	84.6296
18	R0	{1.21884, 7.71545, 1.}	{1.14525, 2.80753, 0.32}	{{1.06738, 2.19254, 0.}, {1.25474, 3.26445, 1.}}	{7.14855, 63.6116, 72.}	inf	68.	30.329	{9.21815, 58.6566}	55.5705	0.445433	newly	61.	89.37	{21., 179.}	80.3115	0.300869	Reff	1.17923	1.11231	{1.03536, 1.21489}	7.05328	ar	3080.	2143.74	{320., 3732.}	42.0305
19	R0	{1.34879, 6.71464, 0.1}	{1.21039, 2.49379, 0.}	{{1.06738, 2.19254, 0.}, {1.24751, 2.5364, 0.}}	{10.3248, 62.8605, 100.}	inf	96.	52.4622	{10.5528, 100.968}	51.3011	0.418861	newly	111.	185.23	{42., 339.}	97.955	0.316231	Reff	1.29578	1.16804	{1.03086, 1.20883}	9.90877	ar	4585.	3083.38	{1107., 4046.}	32.8899
20	R0	{1.37284, 5.75036, 0.1}	{1.19775, 2.8633, 0.06}	{{1.06738, 2.19254, 0.}, {1.25474, 3.26445, 0.}}	{13.459, 50.2067, 150.}	inf	46.	27.0015	{6.37924, 45.4025}	45.8309	0.35216	newly	83.	82.81	{17., 161.}	50.9277	0.283466	Reff	1.34758	1.17796	{1.04911, 1.23597}	13.3044	ar	4750.	2783.58	{192., 4132.}	42.8615
21	R0	{1.29938, 5.36419, 0.1}	{1.37938, 4.65764, 0.3}	{{1.06738, 2.19254, 0.}, {2.01498, 14.4607, 2.}}	{19.2892, 68.2242, 368.}	inf	36.	44.3758	{7.15993, 93.2538}	84.1034	0.349501	newly	59.	91.34	{20., 178.}	88.1356	0.311651	Reff	1.28145	1.35704	{1.05369, 1.97625}	18.9008	ar	4337.	3744.51	{756., 8158.}	43.3555
22	R0	{1.31882, 7.47626, 0.1}	{1.55033, 10.0491, 1.39}	{{1.06738, 2.19254, 0.}, {1.93882, 15.8893, 3.}}	{27.913, 86.1, 1366.}	inf	67.	108.749	{19.9309, 185.898}	101.966	0.381543	newly	68.	97.09	{56., 146.}	53.8382	0.193846	Reff	1.28479	1.50168	{1.04401, 1.86668}	27.0063	ar	3977.	5373.75	{1345., 7856.}	67.1479
23	R0	{1.26215, 3.46375, 0.1}	{1.23449, 4.6465, 0.41}	{{1.06738, 2.19254, 0.}, {1.44762, 6.00596, 1.}}	{10.5437, 49.3745, 466.}	inf	5.	13.2262	{3.36161, 23.9273}	177.366	0.394062	newly	32.	27.52	{7., 53.}	56.625	0.331272	Reff	1.25672	1.2281	{1.05949, 1.43866}	10.5313	ar	3607.	2676.16	{54., 5661.}	53.3368
24	R0	{1.16712, 4.19464, 1.}	{1.82122, 13.0034, 1.93}	{{1.44485, 6.00596, 0.}, {2.1968, 19.9579, 3.}}	{56.0441, 210.262, 119.}	inf	63.	148.493	{70.8685, 238.181}	139.237	0.354064	newly	109.	99.36	{74., 126.}	16.9174	0.0818609	Reff	1.13853	1.75972	{1.40745, 2.1049}	54.5612	ar	3000.	7225.	{5468., 8466.}	140.833
25	R0	{1.22751, 3.34517, 0.1}	{2.13374, 9.55529, 0.7}	{{1.32705, 4.00571, 0.}, {2.91001, 13.7324, 2.}}	{74.5262, 187.512, 702.}	inf	2.	11.486	{2.71385, 19.8655}	476.922	0.678779	newly	9.	16.84	{3., 36.}	118.	0.347449	Reff	1.22628	2.12946	{1.32563, 2.90232}	74.352	ar	3086.	7324.47	{2869., 9312.}	154.667
26	R0	{1.18747, 5.88083, 1.}	{1.06914, 2.19538, 0.03}	{{1.06738, 2.19254, 0.}, {1.06738, 2.19254, 0.}}	{10.0662, 62.6689, 99.}	inf	134.	42.6993	{10.9256, 78.4562}	68.1348	0.598205	newly	150.	136.38	{26., 253.}	47.7333	0.28262	Reff	1.11017	1.0093	{1.00368, 1.01103}	9.20683	ar	3614.	1357.62	{569., 2045.}	62.4344
27	R0	{1.29023, 4.76202, 0.1}	{1.11076, 2.87999, 0.36}	{{1.06738, 2.19254, 0.}, {1.24751, 2.5364, 1.}}	{16.2481, 61.9971, 438.}	inf	32.	24.9489	{3.57147, 50.4201}	53.9488	0.377894	newly	65.	76.63	{10., 147.}	67.6769	0.339329	Reff	1.26391	1.08873	{1.04417, 1.22312}	16.1319	ar	4245.	1481.66	{192., 3786.}	70.4226
28	R0	{1.2258, 12.9056, 3.}	{1.10392, 5.31224, 0.01}	{{1.10202, 5.30671, 0.}, {1.10202, 5.30671, 0.}}	{9.9428, 58.8377, 99.6667}	inf	157.	52.5057	{29.2362, 72.2208}	66.5569	0.504403	newly	86.	68.23	{36., 95.}	27.1977	0.160066	Reff	1.08814	0.991484	{0.987604, 0.993352}	8.88295	ar	3639.	1776.52	{1295., 2370.}	51.1811
29	R0	{1.34702, 6.29049, 0.1}	{2.08473, 13.3676, 0.99}	{{1.44485, 6.00596, 0.}, {2.76795, 19.6041, 2.}}	{55.058, 115.733, 948.}	inf	78.	162.738	{81.4296, 253.901}	113.686	0.302024	newly	109.	111.62	{76., 144.}	17.7982	0.0777263	Reff	1.31765	2.01892	{1.417, 2.65451}	53.502	ar	4560.	7807.27	{5585., 9199.}	72.1577
30	R0	{1.37931, 6.78741, 1.}	{1.23695, 3.33619, 0.06}	{{1.06738, 2.19254, 0.}, {1.28596, 3.98513, 0.}}	{10.321, 50.8474, 98.}	inf	121.	65.1445	{30.7776, 99.9098}	46.5516	0.315349	newly	153.	164.61	{78., 259.}	36.8431	0.164248	Reff	1.31324	1.18456	{1.02615, 1.23226}	9.79869	ar	4667.	3441.41	{2160., 4164.}	26.2608
31	R0	{1.16062, 4.47529, 0.1}	{2.33395, 6.75546, 0.21}	{{1.65456, 4.02511, 0.}, {2.91001, 10.1535, 1.}}	{101.256, 57.2587, 278.}	inf	8.	29.074	{8.4767, 44.3574}	267.598	0.519891	newly	12.	71.32	{24., 124.}	495.833	0.692554	Reff	1.1576	2.32265	{1.64557, 2.89757}	100.804	ar	3037.	8312.17	{6503., 9328.}	174.96
32	R0	{1.37647, 18.7122, 2.}	{1.21632, 8.70736, 3.94}	{{1.21725, 8.76728, 4.}, {1.21725, 8.76728, 4.}}	{11.6346, 53.4669, 99.}	inf	284.	150.741	{129.748, 170.879}	46.9221	0.277605	newly	111.	123.6	{105., 140.}	14.1982	0.056718	Reff	1.17055	1.05057	{1.04858, 1.05379}	10.2502	ar	4824.	3283.52	{2887., 3646.}	31.9337
33	R0	{1.35084, 6.08841, 0.1}	{1.26992, 4.41215, 0.39}	{{1.06738, 2.19254, 0.}, {1.40191, 5.7404, 1.}}	{13.336, 57.6442, 450.}	inf	31.	30.6698	{5.97412, 55.7248}	60.1132	0.312168	newly	44.	65.41	{15., 121.}	77.6136	0.280424	Reff	1.3326	1.25236	{1.05313, 1.38376}	13.1885	ar	4759.	3259.15	{252., 4660.}	42.6915
34	R0	{1.36912, 6.316, 1.}	{2.02156, 13.6485, 2.03}	{{2.01902, 13.4708, 2.}, {2.04423, 14.1631, 2.}}	{48.0949, 117.4, 105.}	inf	47.	191.895	{161.163, 233.323}	308.721	0.603664	newly	61.	125.34	{99., 150.}	105.475	0.306981	Reff	1.33681	1.94424	{1.93332, 1.97265}	45.8777	ar	4537.	7955.84	{7826., 8174.}	76.9248
35	R0	{1.35831, 7.09038, 1.}	{1.4703, 6.59519, 1.11}	{{1.06738, 2.19254, 0.}, {2.01498, 14.4607, 3.}}	{22.9942, 73.6708, 137.}	inf	102.	106.667	{16.6851, 231.828}	75.4657	0.412473	newly	115.	148.16	{60., 216.}	49.6348	0.213541	Reff	1.31498	1.41977	{1.04238, 1.92361}	22.0721	ar	4667.	4695.96	{1347., 7998.}	48.5142
36	R0	{1.28315, 5.89938, 0.1}	{1.90415, 9.22838, 0.72}	{{1.06738, 2.5149, 0.}, {2.93371, 18.1089, 2.}}	{53.3985, 100.293, 718.}	inf	88.	112.42	{16.9514, 231.287}	81.1011	0.368188	newly	139.	119.2	{48., 180.}	30.1295	0.176697	Reff	1.25672	1.855	{1.05399, 2.81502}	52.2849	ar	4460.	6257.56	{2823., 9358.}	66.0072
37	R0	{1.19303, 2.75113, 0.1}	{1.19167, 2.43738, 0.01}	{{1.06738, 2.19254, 0.}, {1.25474, 3.07157, 0.}}	{9.36541, 15.5408, 108.}	inf	7.	24.2633	{4.47091, 49.3886}	260.919	0.500304	newly	22.	95.4	{24., 178.}	346.909	0.578304	Reff	1.1848	1.1813	{1.05812, 1.23964}	9.26733	ar	3142.	2397.78	{109., 4108.}	53.2896
38	R0	{1.36033, 7.8088, 1.}	{2.18134, 15.7767, 2.9}	{{2.01498, 14.4607, 2.}, {2.24606, 16.668, 3.}}	{60.354, 102.038, 190.}	inf	85.	218.021	{149.471, 280.439}	156.495	0.392996	newly	88.	121.59	{85., 157.}	39.8295	0.137735	Reff	1.32551	2.09536	{1.94473, 2.15847}	58.0797	ar	4941.	8340.75	{7915., 8635.}	68.8069
39	R0	{1.27353, 3.70845, 0.1}	{1.216, 2.51103, 0.04}	{{1.06738, 2.19254, 0.}, {1.25474, 3.07157, 0.}}	{5.81594, 32.2891, 136.}	inf	37.	30.9147	{6.51051, 59.0582}	45.4875	0.300135	newly	69.	122.38	{23., 255.}	108.029	0.343889	Reff	1.24908	1.19335	{1.04895, 1.23088}	5.77287	ar	3725.	3028.32	{278., 4143.}	27.7444
40	R0	{1.19983, 12.1486, 0.1}	{1.11534, 4.92784, 0.26}	{{1.06738, 2.5149, 0.}, {1.13726, 6.0871, 1.}}	{7.04169, 59.437, 308.}	inf	164.	60.4535	{23.8906, 89.7413}	63.1381	0.485145	newly	86.	84.89	{48., 133.}	28.4535	0.136966	Reff	1.07625	1.01197	{0.972671, 1.03144}	5.97271	ar	3619.	1929.69	{1141., 2679.}	46.6789
41	R0	{1.20935, 2.67169, 0.1}	{1.8154, 4.42238, 0.03}	{{1.25474, 2.5364, 0.}, {2.49133, 6.2492, 0.}}	{50.3489, 69.3157, 124.}	inf	13.	35.4811	{11.5327, 66.0403}	183.429	0.409799	newly	45.	120.24	{41., 238.}	177.511	0.382222	Reff	1.20379	1.80248	{1.24742, 2.47282}	49.9679	ar	3429.	6691.92	{3761., 8915.}	101.21
42	R0	{1.23313, 4.74781, 1.}	{1.0946, 2.40415, 0.09}	{{1.06738, 2.19254, 0.}, {1.22544, 3.07157, 0.}}	{11.9547, 49.3629, 95.}	inf	49.	34.7027	{9.78559, 62.1527}	42.0006	0.295881	newly	79.	111.01	{29., 197.}	75.6329	0.297825	Reff	1.18196	1.05071	{1.02147, 1.17791}	11.8294	ar	3835.	1491.78	{419., 3378.}	61.6829
43	R0	{1.26105, 2.39713, 0.1}	{1.19659, 2.36537, 0.04}	{{1.06738, 2.07736, 0.}, {1.39162, 2.5364, 0.}}	{8.90571, 9.78871, 134.}	inf	6.	9.83191	{2.01304, 19.6682}	98.1141	0.31215	newly	32.	46.35	{7., 106.}	102.031	0.377477	Reff	1.25298	1.18845	{1.05951, 1.37929}	8.91077	ar	3723.	2122.32	{70., 4647.}	55.1501
44	R0	{1.36948, 8.88006, 0.1}	{1.18313, 2.87479, 0.11}	{{1.06738, 2.19254, 0.}, {1.25474, 3.26445, 0.}}	{13.7218, 67.6265, 190.}	inf	75.	33.8959	{8.19815, 59.2705}	55.0588	0.457221	newly	68.	100.57	{23., 187.}	76.8676	0.288356	Reff	1.33689	1.15979	{1.04485, 1.23202}	13.3662	ar	4437.	2714.99	{315., 3985.}	39.2916
45	R0	{1.34882, 3.3791, 0.1}	{2.01056, 11.3751, 0.58}	{{1.22544, 5.15387, 0.}, {2.83858, 18.6082, 2.}}	{52.9065, 237.382, 600.}	inf	2.	4.80599	{1.51145, 9.37181}	151.408	0.335596	newly	4.	7.55	{1., 20.}	152.25	0.482622	Reff	1.34828	2.00911	{1.22423, 2.83669}	52.8621	ar	4422.	5967.13	{6., 9285.}	80.9767
46	R0	{1.23774, 4.34917, 0.1}	{1.94856, 6.79308, 0.32}	{{1.47488, 4.00571, 0.}, {2.68687, 11.6897, 1.}}	{57.4288, 61.6407, 372.}	inf	31.	82.6856	{25.4291, 144.232}	177.924	0.419354	newly	58.	139.74	{62., 219.}	148.655	0.382627	Reff	1.22252	1.91289	{1.44982, 2.62269}	56.4718	ar	3685.	7381.21	{5549., 9155.}	100.474
47	R0	{1.34327, 4.78811, 0.1}	{2.08716, 9.70184, 0.67}	{{1.25474, 3.26445, 0.}, {2.68803, 16.884, 2.}}	{57.7923, 113.789, 678.}	inf	9.	47.4205	{13.4959, 76.2857}	432.307	0.665679	newly	13.	54.25	{24., 81.}	320.077	0.568509	Reff	1.33642	2.06749	{1.24917, 2.65948}	57.1424	ar	4517.	7448.26	{3959., 9182.}	75.1751
48	R0	{1.24366, 4.25814, 0.1}	{1.19896, 3.08303, 0.09}	{{1.06738, 2.19254, 0.}, {1.28596, 3.98513, 0.}}	{6.35324, 27.5969, 172.}	inf	15.	19.4404	{5.52232, 32.7935}	67.0032	0.273679	newly	19.	53.21	{9., 101.}	197.526	0.436564	Reff	1.23073	1.18594	{1.05508, 1.27255}	6.32891	ar	3678.	2616.8	{127., 4162.}	37.5753
49	R0	{1.36017, 7.5415, 1.}	{1.09768, 2.40713, 0.11}	{{1.06738, 2.19254, 0.}, {1.22544, 3.07157, 0.}}	{19.2986, 68.0816, 91.}	inf	84.	46.6965	{14.0951, 86.023}	48.2327	0.363494	newly	104.	139.91	{43., 253.}	66.8365	0.258406	Reff	1.284	1.04029	{1.00759, 1.16201}	18.9806	ar	4178.	1705.86	{640., 3271.}	59.3289
50	R0	{1.25333, 6.63578, 1.}	{1.06748, 2.23674, 0.09}	{{1.06738, 2.19254, 0.}, {1.06738, 2.19254, 0.}}	{14.8282, 66.2927, 91.}	inf	101.	49.1512	{9.54779, 87.1191}	52.5245	0.431301	newly	117.	148.67	{27., 263.}	69.1026	0.313698	Reff	1.14855	0.983777	{0.978864, 0.987904}	14.3463	ar	3401.	1424.94	{800., 2012.}	58.1023
